# Heavy Metals and Related Human Health Risk Assessment for River Waters in the Issyk−Kul Basin, Kyrgyzstan, Central Asia

**DOI:** 10.3390/ijerph17103506

**Published:** 2020-05-17

**Authors:** Wen Liu, Long Ma, Yaoming Li, Jilili Abuduwaili, Salamat Abdyzhapar uulu

**Affiliations:** 1 State Key Laboratory of Desert and Oasis Ecology, Xinjiang Institute of Ecology and Geography, Chinese Academy of Sciences, Urumqi 830011, China; liuwen@ms.xjb.ac.cn (W.L.); lym@ms.xjb.ac.cn (Y.L.); jilil@ms.xjb.ac.cn (J.A.); 2 Research Center for Ecology and Environment of Central Asia, Chinese Academy of Sciences, Urumqi 830011, China; salamattalas18@gmail.com; 3 University of Chinese Academy of Sciences, Beijing 100049, China; 4 Institute of Geology, National Academy of Sciences of Kyrgyzstan, Bishkek 720461, Kyrgyzstan

**Keywords:** heavy metals, human health risk, river waters, Issyk−Kul Basin, Central Asia

## Abstract

The water resources of Central Asia play an important role in maintaining the fragile balance of ecosystems and the sustainable development of human society. However, the lack of research on the heavy metals in river waters has a far−reaching influence on public health and the sustainable development in Central Asia. In order to reveal the possible sources of the heavy metals and to assess the associated human health risks, thirty−eight water samples were collected from the rivers of the Issyk−Kul Basin during the period with low river flow (May) and the period with high river flow (July and August), and the hydrochemical compositions and major ions of heavy metals were analyzed. No changes in hydrochemical facies were observed between the two periods and the river water type was calcium bicarbonate. Carbonate dissolution and silicate weathering controlled the variation of cations and anions in river waters from the Issyk−Kul Basin. There were some differences in the sources of heavy metals in water bodies between the two periods. During the period with low river flow, heavy metals (Cr) were closely clustered with major ions, indicating that they were mainly affected by water–rock interactions. During the period with high river flow, all heavy metals studied in this paper had different sources of major ions, and the heavy metals maybe influenced by human activities. From the human health risk assessment, the hazard quotients for all samples were less than 1, reflecting that there was no noncarcinogenic risk in the river waters of the Issyk−Kul Basin during the two sampling periods. However, the water samples with carcinogenic risk of arsenic exceeding the threshold (10^−4^) accounted for 21.1% of the total, indicating that there were some certain carcinogenic hazards for human health via water drinking with direct oral ingestion. The results are of certain significance for the utilization and protection of water resources in the basin as well as the protection of public health.

## 1. Introduction

The water resources of Central Asia play an important role in maintaining the fragile balance of ecosystems and the sustainable development of human society [1,2,3]. The shortage of water resources in Central Asia represented by the Aral Sea crisis has aroused concern worldwide [4,5]. At present, most studies focus on water quantity and water management under the influences of recent strong fluctuations in climate and increasing human activity in Central Asia [6,7,8,9,10]. For water security in Central Asia, it is also necessary to carry out research focused on water quality [11,12]. The problem of water pollution caused by industrial, agricultural and domestic wastewater is becoming increasingly serious; currently, the impact of water quality on human health in Central Asia had begun to be highlighted [13,14,15]. Compared with the comprehensive study of water quantity and its influencing factors, research on water quality and its influencing factors needs to be strengthened. Especially, heavy metal, as one of the pollutants in river waters, cause severe threats to humans and the environment [16]. Heavy metal contamination will induce human health risks for direct drinking and via food crop consumption with water irrigation [17,18]. On the one hand, river water resources in Central Asia are mainly used for farmland irrigation [19,20]; on the other hand, according to the field investigation, limited to the level of economic development, some people use surface river water as a direct source of drinking water. Therefore, it is of great practical significance to study heavy metals and their health risks in the rivers of Issyk−Kul Basin.

The Issyk−Kul Basin (22,080 km^2^) is the largest basin in the Tien Shan Mountains [21], which covers 11.1% of the country’s land area (198,500 km^2^), and the Issyk−Kul Lake (6236 km^2^) covers 28.2% of the basin’s surface area [22]. For the Issyk−Kul Basin, the soils in the basin are mainly composed of Haplic Kastanozems and Mollic Leptosols [23]. The hydrochemical composition of rivers not only affects the safety of agricultural irrigation and drinking water but also directly affects the ecological security of Issyk−Kul Lake. However, remarkably little is known about the hydrochemical composition of the basin waters, especially regarding heavy metals. At present, uranium pollution in natural waters [24,25] and heavy metals in surface soils [26] of Issyk−Kul Basin has attracted attention, but other heavy metals in river waters which have potential effects on human health are still uninvestigated. As we know, the problems from heavy metal water pollution [27,28] are becoming increasingly serious, and it is very important to evaluate the sources of heavy metals and perform a related human risk assessment for the river water of the Issyk−Kul Basin.

Based on the above considerations, 38 river water samples were collected from the Issyk−Kul Basin during the period with low river flow (*n = 19*) and the period with high river flow (*n = 19*), and the heavy metals and major ions in river waters were analysed in this study. Specifically, this study aimed to reveal the characteristics of heavy metals in river waters and identify their temporal differences to perform a human health risk assessment. The results are of certain significance for the utilization and protection of water resources in the basin as well as the protection of public health.

## 2. Materials and Methods

### 2.1. Sampling and Analysis

**Figure 1:**
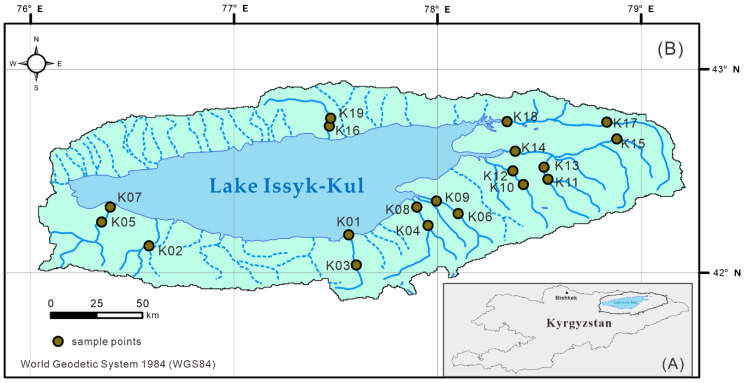
Location map of the Issyk−Kul Basin in Kyrgyzstan (**A**) and the distribution of the river water samples (**B**).

In the study area of the Issyk−Kul Basin, samples were collected from 19 sampling points during the low river flow period (May 2017), and 19 were collected from the same points during the high−flow period (July and August 2017) (Table 1 and Figure 1), for a total of 38 samples. Due to the influence of mountain topography, the precipitation in Issyk−Kul Lake basin varies greatly from north to south, and the precipitation in the south is significantly higher than that in the north [29], which leads to the long−term interruption of the rivers in the north side of the basin. River water samples that were rinsed three times in situ were collected in 1.5−L polyethylene terephthalate bottles. The subsamples were filtered through a 0.45−μm filter and stored in a polyethylene tube for subsequent laboratory measurements. Before sampling and analysis of cations and heavy metals, samples were mixed with 10% HNO_3_ and three drops of HNO_3_ (65%) for subsample acidification (pH < 2). A HI 9828 multiparameter water quality meter (Hanna Instruments, Villafranca Padovana, Italy) was used to measure pH, electrical conductivity (EC) and total dissolved solids (TDS) in situ. HCO_3_ and CO_3_ were determined by potentiometric titration using a G20 compact titrator (Mettler Toledo AG, Greifensee, Switzerland). The major ions were measured by a Dionex ICS 900 ionic chromatography system (Thermo Fisher Scientific Inc., Waltham, MA, USA) [30]. The charge balance error (CBE) percentage [31,32] was used to evaluate the accuracy of the results for the main cations and anions, and the CBE was less than 5%. The contents of dissolved heavy metals in river waters, including zinc (Zn), copper (Cu), cadmium (Cd), lead (Pb), total chromium (Cr) and arsenic (As), were determined by inductively coupled plasma mass spectrometry with an Agilent 8800 system (Agilent Technologies, Santa Clara, CA, USA). The blank solution of 1 % nitric acid was measured for 21 consecutive times, and the concentration corresponding to 4.6 standard deviations was used as the detection limit for Zn (0.003 μg L^−1^), Cu (0.003 μg L^−1^), Cd (0.003 μg L^−1^), Pb (0.001 μg L^−1^), Cr (0.02 μg L^−1^), and As (0.006 μg L^−1^). The standard sample was measured 12 times and its precision and accuracy was calculated. Experiments show that the precision of heavy metal elements is between 0.74 % and 3.17 %. The aforementioned analyses were performed at the Research Center for Ecology and the Environment of Central Asia (Bishkek), Kyrgyzstan.

### 2.2. Human Health Risk Assessment

Direct oral ingestion is the main pathway of heavy metal exposure from aqueous systems. The human health risk assessment for noncarcinogens was calculated using Equations (1) and (2) [27,33,34,35]. If the noncarcinogenic hazard quotient (HQ) < 1, no noncarcinogenic risks are suggested; otherwise, noncarcinogenic effects exist [35,36].

(1) $$ADD=C_{h}\times \frac{IngR\times EF\times ED}{BW\times AT}$$

(2) HQ=ADD/RfD

where HQ: noncarcinogenic hazard quotient; ADD: average daily dose of exposure through water absorption; **C_h_**: concentration of heavy metals in river waters, mg L^−1^; IngR: ingestion rate, 2 L/day [27]; EF: exposure frequency, 350 day/year [27]; ED: exposure duration, 70 years [33]; BW: body weight, 70 kg [27]; AT: average time, 25550 days [33]; RfD: reference value for heavy metals [37].

Risks from carcinogens were evaluated by Equation (3) [38], and the acceptable range of CR by the USEPA was 10^−6^ to 10^−4^ [39].

(3) CR=ADD×CSF

where **ADD:** average daily dose of exposure through water absorption; CSF: cancer slope factor [37].

## 3. Results

For the river waters of the Issyk−Kul Basin shown in Table 2 and Table 3, the concentration of Zn varied between 0.634 and 9.75 μg L^−1^, with a mean of 5.43 μg L^−1^, during the period with low river flow, and the concentration of Zn varied between 0.359 and 26.3 μg L^−1^, with a mean of 9.60 μg L^−1^, during the period with high river flow. The Cu concentration varied between 1.08 and 8.51 μg L^−1^, during the period with low river flow, with a mean of 3.64 μg L^−1^, and it varied between 0.30 and 12.5 μg L^−1^ during the period with high river flow, with a mean of 4.23. The Pb level varied between 0 and 2.19 μg L^−1^, with a mean of 1.01 μg L^−1^, during the period with low river flow, and from 0 to 4.06 μg L^−1^, with a mean of 1.52 μg L^−1^, during the period with high river flow. As varied between 0.461 and 2.08 μg L^−1^, with a mean of 1.17, during the period with low river flow, and from 0.134 to 3.03μg L^−1^, with a mean of 1.25, during the period with high river flow. The Cr value varied between 0.01 and 0.05 mg L^−1^, with a mean of 0.03 mg L^−1^, during the period with low river flow, and between 0.01 and 0.07 mg L^−1^, with a mean of 0.036 mg L^−1^, during the period with high river flow.

During the period with low river flow (May 2017), the pH varied from 7.55 to 8.94, with a mean of 8.20. During the period with high river flow (July and August 2017), the pH varied from 7.79 to 8.84, with a mean of 8.25. The EC ranged between 60 and 299 μS cm^−1^, with a mean of 144.53 μS cm^−1^, during the period with low river flow, and from 71 to 352 μS cm^−1^, with a mean of 170 μS cm^−1^, during the period with high river flow. The TDS ranged from 110 to 355 mg L^−1^, with a mean of 204 mg L^−1^, during the period with low river flow, and the TDS varied from 119 to 392 mg L^−1^, with a mean of 217 mg L^−1^, during the period with high river flow.

**Figure 2:**
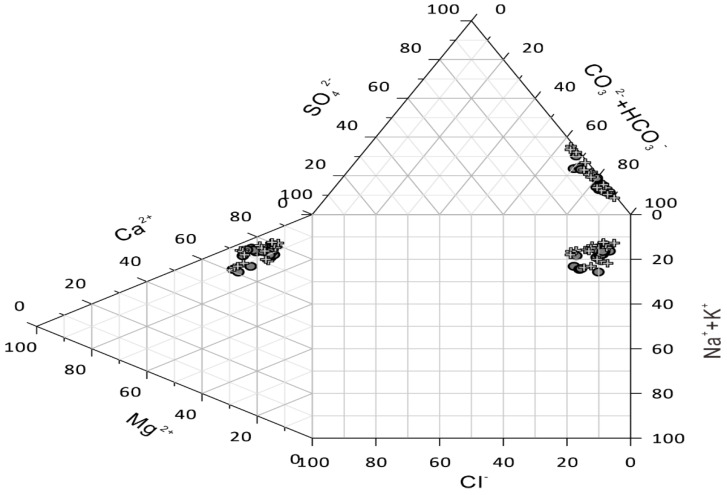
Durov diagram for the water samples from the Issyk−Kul Basin. The grey dots correspond to the samples during the period with low river flow (L, *n = 19*) and the black crosses correspond to the period with high river flow (H, *n = 19*).

As seen from the table showing statistics on the concentration of major ions in the river waters, HCO_3_^−^ was found in the highest concentration. No changes in hydrochemical facies were observed between the two periods. As seen in the Durov diagrams [40,41], the river water type was calcium bicarbonate (Figure 2).

## 4. Discussion

**Figure 3:**
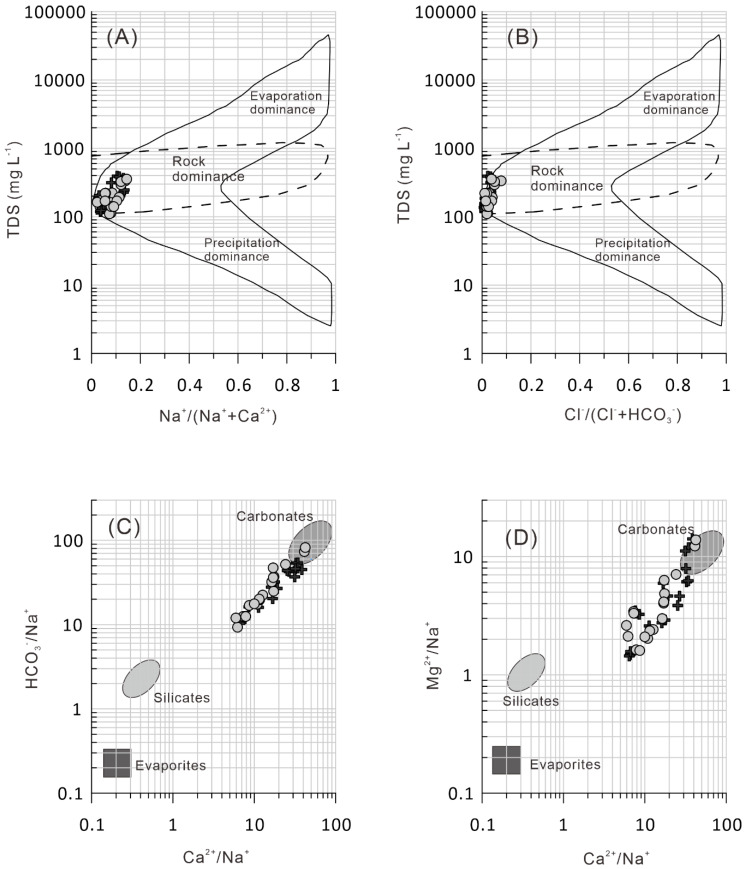
Gibbs plots (**A**,**B**) and mixing diagrams (**C**,**D**) for the river water samples from the Issyk−Kul Basin. The grey dots correspond to the period with low river flow (*n = 19*) and the black crosses correspond to the period with high river flow (*n = 19*).

In order to determine the detailed factors affecting water chemistry, Gibbs diagrams [42,43,44] and mixing diagrams [42,45,46,47] were used. For the Gibbs and mixing diagrams, the concentrations of major ions were transformed to milliequivalents per liter [48] as seen in Figure 3. This suggests that river samples plotted in the dominant area are dominated by rock, which suggests that major ions in river water from the Issyk−Kul Basin are mainly controlled by the process of water–rock interactions. The river samples were located between the two end−members of carbonate and silicate (Figure 3). Figure 3 shows that river water samples from the Issyk−Kul basin had high Ca/Na ratios, indicating that the importance of carbonate dissolution is greater than that of silicate weathering.

**Figure 4:**
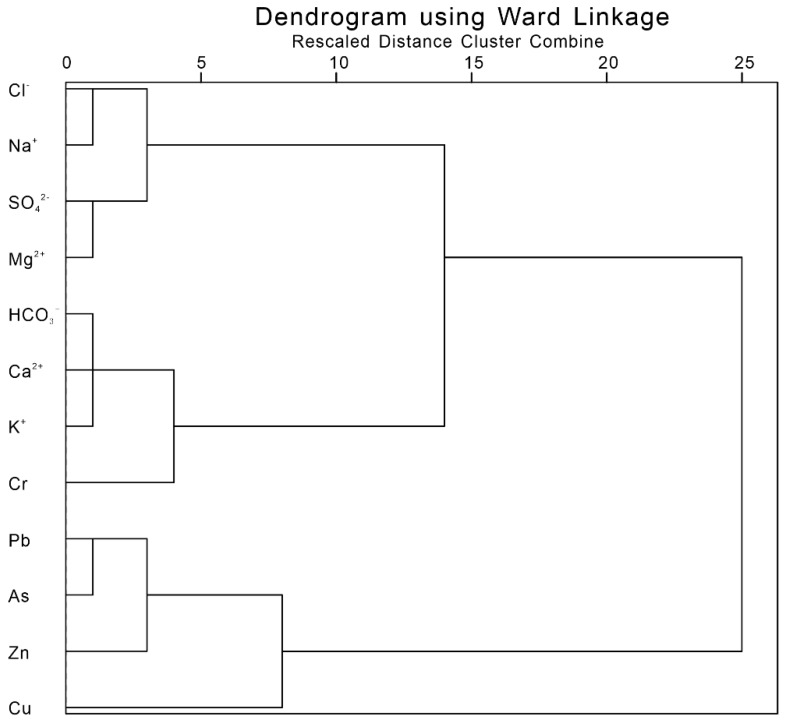
Hierarchical clustering analysis of major ionic and dissolved heavy metal elements in the river waters from Issyk−Kul Lake Basin during the period with low river flow.

**Figure 5:**
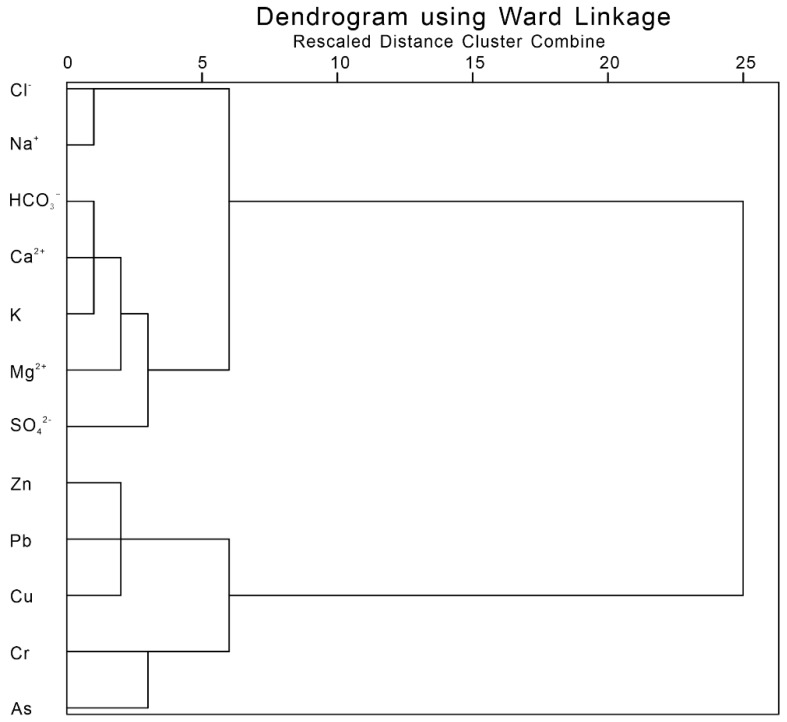
Hierarchical clustering analysis of major ionic and dissolved heavy metal elements in the river waters from Issyk−Kul Lake Basin during the period with high river flow.

The statistical characteristics of hydrochemical data including major ions and heavy metals (Table 2) cannot explain the relationship between various chemical proxies, so we used the hierarchical cluster analysis (HCA) [49] to clarify the possible influences on heavy metals in the river waters of the Issyk−Kul Basin [50,51,52,53]. The chemical compositions of the river waters were analysed at different periods using HCA. The results showed that there were some differences in the sources of heavy metals in water bodies between the two periods. During the period with low river flow, the heavy metal element Cr was closely related to calcium, bicarbonate, and potassium ions (Figure 4). This indicates that the heavy metal elements were mainly affected by the interaction between water and rock and mainly arise from the natural weathering of rock. However, the relationship between other heavy metals (Pb, Zn, Cu, and As) and the major ions was more distant, which reflects different ion sources and suggests effects from human activities (Figure 4). For the period with high river flow, heavy metals were clustered far from the major ions, reflecting different natural origins of these major ions (Figure 5). It can be concluded that heavy metals are affected by different factors during different hydrological periods.

**Figure 6:**
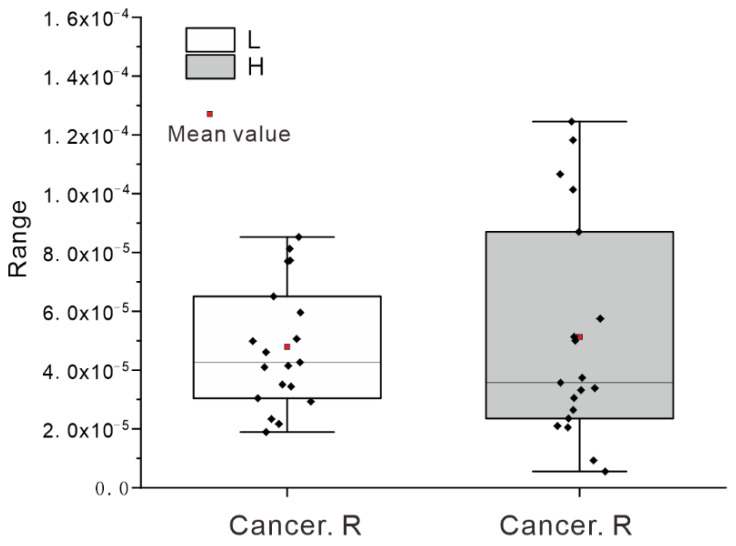
Carcinogenic risks (Cancer. R) for the arsenic in the river waters of the Issyk−Kul Basin during the period with high river flow (H, *n = 19*) and the period with low river flow (L, *n = 19*).

The Issyk−Kul Basin is an important international tourist area, and the number of international tourists visiting this area can be quite high. The noncarcinogenic and carcinogenic risks related to the water in this area have important practical significance in public health. From the calculated results (Table 4), the hazard quotient for noncarcinogenic risk was less than one during the two sampling periods, reflecting that no noncarcinogenic risk was posed by heavy metals in the river waters of the Issyk−Kul Basin; however, the heavy metal Cr was present in concentrations close to the threshold value, which needs more attention. From the carcinogenic risk index, the maximum value of the heavy metal As was more than 10^−4^ (maximum was 1.25 × 10^−4^) during the period with high river flow, and the carcinogenic risk exceeding the threshold account for 21.1% of the total samples, which indicates that there is a certain carcinogenic risk to human health (Figure 6). Although it is not more than 10^−4^, during the period with low river flow, it is also close to the threshold and needs a high degree of attention.

Arsenic is a Class−A human carcinogen [54], and the exposure to arsenic through direct drinking of arsenic−contaminated water or consumption of arsenic−contaminated edible crops is considered a worldwide life−threatening problem [55,56,57]. Through human health risk assessment, the heavy metal of Arsenic in river waters has shown some harm to human health. Based on this, the exact material source of arsenic and its migration and transformation in water bodies requires in−depth discussion. However, based on the existing data and other issues, it is not possible to elaborate on the above issues, and we need to carry out in−depth research in the next step. 

Although the risks of noncarcinogenic and carcinogenic effects are low, the heavy metals in river waters may be affected by human activities, especially during wet periods with high river flow. As the Issyk Lake area is an internationally famous area for tourism and one of the main agricultural and livestock production areas in Kyrgyzstan, environmental protection of this area is very important. Thus, the treatment and discharge of sewage and the protection of the aquatic environment need to be given enough attention to avoid repeating the traditional pattern of experiencing pollution problems before solutions are implemented.

## 5. Conclusions

Based on the concentrations of major ions and heavy metals in river water samples, the possible sources of heavy metals in the Issyk−Kul Basin, Kyrgyzstan, Central Asia, and related human health risks were revealed. The results were as follows:

Carbonate dissolution and silicate weathering accounted for the variation in the major ions of river waters from the Issyk−Kul Basin. No changes in hydrochemical facies were observed between the two sampling periods, and all water samples belong to the type of calcium bicarbonate. 

During the period with low river flow, the heavy metal Cr was mainly affected by water–rock interactions, and the sources of other heavy metals were different from the major ions. During the period with high river flow, all heavy metals studied in this paper had different sources of major ions, and the heavy metals were maybe influenced by human activities.

Based on the human health risk, the hazard quotients were less than 1, reflecting no noncarcinogenic risk for the heavy metals in the studied river waters. However, the water samples with carcinogenic risk for arsenic exceeding the threshold (10^−4^) account for 21.1% of the total, indicating that there was a certain carcinogenic risk in river water in this area.

## Figures and Tables

**Table 1:** Geographic information for sampling points in the Issyk−Kul Basin during the period with low river flow (L) and period with high river flow (H).

NO	Latitude (°N)	Longitude (°E)	Elevation (m)	River	Sampling Date (L)	Sampling Date(H)
K01	42.03494	77.60447	2210	Barskoon	21 May 2017	05 August 2017
K02	42.12865	76.58558	1935	Ak-Terek	23 May 2017	06 August 2017
K03	42.18226	77.56650	1615	Ak-Terek	21 May 2017	05 August 2017
K04	42.22949	77.95718	1950	Juuku	20 May 2017	04 August 2017
K05	42.24544	76.35191	1850	Tuura-Suu	23 May 2017	07 August 2017
K06	42.28658	78.10559	2023	Chon Kyzyl-Suu	19 May 2017	03 August 2017
K07	42.31846	76.39488	1664	Tuura-Suu	23 May 2017	07 August 2017
K08	42.31882	77.90189	1693	Juuku	20 May 2017	04 August 2017
K09	42.34861	77.99841	1736	Chon Kuzul-Suu	19 May 2017	03 August 2017
K10	42.43021	78.42594	1959	Karakol	18 May 2017	02 August 2017
K11	42.45497	78.54606	1976	Ak-Suu	18 May 2017	01 August 2017
K12	42.49698	78.37384	1722	Karakol	18 May 2017	02 August 2017
K13	42.51473	78.52718	1774	Ak-Suu	18 May 2017	01 August 2017
K14	42.59347	78.38598	1634	Jurgalan	17 May 2017	31 July 2017
K15	42.65318	78.88481	1903	Jurgalan	17 May 2017	31 July 2017
K16	42.71734	77.47164	1745	Chon Ak-Suu	15 May 2017	29 July 2017
K17	42.73564	78.83572	1857	Tyup	16 May 2017	30 July 2017
K18	42.73828	78.34636	1623	Tyup	16 May 2017	30 July 2017
K19	42.75582	77.47808	1862	Chon Ak−Suu	15 May 2017	29 July 2017

**Table 2:** Environmental indicators of river water in the Issyk−Kul Basin during the period with low river flow (L, *n = 19*).

Indicators	Minimum	Maximum	Mean	Median	Standard Deviation	Standard Error
Zn^a^ (μg L^−1^)	0.634	9.75	5.43	5.06	2.13	0.49
Cu^a^ (μg L^−1^)	1.08	8.51	3.64	3.54	2.29	0.526
Pb^a^ (μg L^−1^)	0.01	2.19	1.01	0.9	0.641	0.147
Cr^a^ (mg L^−1^)	0.01	0.05	0.032	0.03	0.011	0.003
As^a^ (μg L^−1^)	0.461	2.08	1.17	1.04	0.496	0.114
pH	7.55	8.94	8.2	8.11	0.472	0.108
TDS (mg L^−1^)	110	355	204	172	71.3	16.3
EC (μS cm^−1^)	60	299	145	115	71.1	16.3
Ca^2+^ (mg L^−1^)	14.7	44.6	25.2	20.9	8.8	2.02
Mg^2+^ (mg L^−1^)	1.7	10.9	4.6	3.33	3.05	0.7
Na^+^ (mg L^−1^)	0.522	8.23	2.86	1.87	2.33	0.534
K^+^ (mg L^−1^)	1.1	2.65	1.5	1.36	0.407	0.093
HCO_3_^−^(mg L^−1^)	78.6	250	140	125	44.8	10.3
SO_4_^2−^ (mg L^−1^)	8.21	53.8	23.8	17.6	13.9	3.19
Cl ^−^(mg L^−1^)	0.926	10	2.75	1.55	2.42	0.556

^a^: Dissolved heavy metals of river waters in the Issyk−Kul Basin.

**Table 3:** Environmental indicators of river water in the Issyk−Kul Basin during the period with high river flow (H, *n = 19*).

Indicators	Minimum	Maximum	Mean	Median	Standard Deviation	Standard Error
Zn^a^ (μg L^−1^)	0.359	26.30	9.60	8.2	6.79	1.56
Cu^a^ (μg L^−1^)	0.297	12.5	4.23	3.09	3.62	0.831
Pb^a^ (μg L^−1^)	0	4.06	1.52	1.32	1.26	0.29
Cr^a^ (mg L^−1^)	0.01	0.07	0.036	0.03	0.015	0.003
As^a^ (μg L^−1^)	0.134	3.03	1.25	0.869	0.889	0.204
pH	7.79	8.84	8.25	8.17	0.311	0.071
TDS (mg L^−1^)	119	392	217	186	89.6	20.6
EC (μS cm^−1^)	71	352	170	138	88	20.2
Ca^2+^ (mg L^−1^)	17.4	53	30.7	27.3	11.9	2.72
Mg^2+^ (mg L^−1^)	1.64	13.4	5.42	4.34	3.46	0.795
Na^+^ (mg L^−1^)	0.744	9.12	3.07	1.27	2.78	0.637
K^+^ (mg L^−1^)	1.1	2.65	1.5	1.36	0.407	0.093
HCO_3_^−^(mg L^−1^)	0.838	2.46	1.5	1.45	0.395	0.091
SO_4_^2−^ (mg L^−1^)	84.3	287	144	111	62.4	14.3
Cl ^−^(mg L^−1^)	6.9	49.2	27.4	21.8	14.2	3.26
Ca^2+^ (mg L^−1^)	0.471	6.37	2.23	1.13	1.9	0.437

^a^: Dissolved heavy metals of river waters in the Issyk−Kul Basin.

**Table 4:** Human health risk assessment including the noncarcinogenic hazard quotients (non. HQ) and carcinogenic risks (Cancer. R) from heavy metals in the river waters of the Issyk−Kul Basin during the period with high river flow (H) and the period with low river flow (L).

Stage	Heavy metals	Minimum ^a^	Maximum ^b^	Mean	Median
H	**Zn (non.HQ)**	3.27 × 10^−5^	2.40 × 10^−3^	8.76 × 10^−4^	7.49 × 10^−4^
	**Cu (non. HQ)**	2.04 × 10^−4^	8.59 × 10^−3^	2.89 × 10^−3^	2.12 × 10^−3^
	**Pb (non. HQ)**	0	3.18 × 10^−4^	1.19 × 10^−4^	1.03 × 10^−4^
	**Cr (non. HQ)**	9.13 × 10^−2^	6.39 × 10^−1^	3.32 × 10^−1^	2.74 × 10^−1^
	**As (non. HQ)**	1.22 × 10^−2^	2.77 × 10^−1^	1.14 × 10^−1^	7.93 × 10^−2^
	**As (Cancer.R)**	5.51 × 10^−6^	1.25 × 10^−4^	5.12 × 10^−5^	3.57 × 10^−5^
L	**Zn (non.HQ)**	5.79 × 10^−5^	8.90 × 10^−4^	4.96 × 10^−4^	4.62 × 10^−4^
	**Cu (non. HQ)**	7.42 × 10^−4^	5.83 × 10^−3^	2.49 × 10^−3^	2.42 × 10^−3^
	**Pb (non. HQ)**	8.05 × 10^−7^	1.72 × 10^−4^	7.90 × 10^−5^	7.04 × 10^−5^
	**Cr (non. HQ)**	9.13 × 10^−2^	4.57 × 10^−1^	2.89 × 10^−1^	2.74 × 10^−1^
	**As (non. HQ)**	4.21 × 10^−2^	1.89 × 10^−1^	1.06 × 10^−1^	9.48 × 10^−2^
	**As (Cancer.R)**	1.89 × 10^−5^	8.53 × 10^−5^	4.79 × 10^−5^	4.27 × 10^−5^

^a^: The minimum value of heavy metals (C_h_, Equation (1)) was shown in Table 2. ^b^: The maximum value of (C_h_, Equation (1)) was shown in Table 2.
